# GWAS on your notebook: fast semi-parallel linear and logistic regression for genome-wide association studies

**DOI:** 10.1186/1471-2105-14-166

**Published:** 2013-05-28

**Authors:** Karolina Sikorska, Emmanuel Lesaffre, Patrick FJ Groenen, Paul HC Eilers

**Affiliations:** 1 Department of Biostatistics, Erasmus MC, Rotterdam, The Netherlands; 2 Departments of Internal Medicine and Epidemiology, Erasmus MC, Rotterdam, The Netherlands; 3 L-Biostat, KU Leuven, Leuven, Belgium; 4 Econometric Institute, Erasmus University, Rotterdam, The Netherlands

## Abstract

**Background:**

Genome-wide association studies have become very popular in identifying genetic contributions to phenotypes. Millions of SNPs are being tested for their association with diseases and traits using linear or logistic regression models. This conceptually simple strategy encounters the following computational issues: a large number of tests and very large genotype files (many Gigabytes) which cannot be directly loaded into the software memory. One of the solutions applied on a grand scale is cluster computing involving large-scale resources. We show how to speed up the computations using matrix operations in pure R code.

**Results:**

We improve speed: computation time from 6 hours is reduced to 10-15 minutes. Our approach can handle essentially an unlimited amount of covariates efficiently, using projections. Data files in GWAS are vast and reading them into computer memory becomes an important issue. However, much improvement can be made if the data is structured beforehand in a way allowing for easy access to blocks of SNPs. We propose several solutions based on the R packages **ff** and **ncdf**.

We adapted the semi-parallel computations for logistic regression. We show that in a typical GWAS setting, where SNP effects are very small, we do not lose any precision and our computations are few hundreds times faster than standard procedures.

**Conclusions:**

We provide very fast algorithms for GWAS written in pure R code. We also show how to rearrange SNP data for fast access.

## Background

For the benefit of readers who are not familiar with genome-wide association studies we provide a brief introduction to this area.

There are many ways to investigate the influence of genes on (human) traits. One of them, genome-wide association studies (GWAS), exploits the fact that strings of DNA contain many small variations, called SNPs which may influence the level of traits or risk of having a disease. Modern micro-array technology makes it possible to measure genotypes of a million SNPs in one go, at a reasonable price, using only one drop of blood. In large epidemiological studies, this has been done for large to very large groups of individuals, for which (many) phenotypes have been measured too. SNPs that are found to be influential may point to relevant genes. This approach has been applied on a grand scale [1]. The number of results published on GWAS is rapidly increasing. The GWAS catalogue includes over 1400 papers on newly discovered important SNPs [2].

Typically, the number of genotyped SNPs is around half a million. However, it is possible to impute the most probable genotypes for real or hypothetical SNPs using spatial correlation on the genome. This way, the number of SNPs analyzed in a GWAS can grow to 2.5 or even 30 million.

The statistical model used in GWAS is rather basic: univariate linear or logistic regression of phenotype on genotypes, for each SNP in turn, correcting for covariates like age, height and gender. Large sample sizes are required to detect very small effects at the very strict “GWA-significance level”, namely 5×10^-8^, the common 0.05 divided by one million (inspired by Bonferroni correction for that many tests). The goal is to find SNPs for which the *p*-value will survive this conservative multiple testing correction.

Dedicated software is available to support those analyzes. Popular examples are: GenABEL [3], PLINK[4], Mach2qtl[5,6] and ProbABEL [7]. Computation times are long. An example from the literature is a GWAS with a continuous trait for 6000 individuals and 2.5 mln SNPs, which on “a regular computer” takes around 6 hours [8]. This time will dramatically increase with larger sample size and/or more SNPs to test. Additionally, logistic regression is more computationally demanding than linear regression. Based on the available published materials, it is actually quite difficult to assess computation times. Usually information about available memory, number of used processors/cores, and the size of the model (the number of covariates) are not provided.

GWAS may be computationally demanding, but the problem is “embarrassingly parallel”, meaning that it can be distributed over as many processors as desired, by simply splitting the work into groups of SNPs. This brute force approach with computing clusters is now being applied broadly, with GRIMP as an example in our institution [8].

We show that huge speed gains can be achieved by simple rearrangements of linear model computations, exploiting fast matrix operations. We call this the “semi-parallel” approach, to set it apart from parallel computation on multiple processors. A similar idea can be found in [9], in the framework of expression quantitative trait loci (eQTL) analysis. That paper focuses on computing *R*^2^ statistics, to get a first insight into a data. We are more ambitious: we want to reproduce very closely the results of “traditional” GWAS software. Present-day GWAS practice is focused on very low *p*-values, regardless of the amount of variance that the SNPs actually explain. Thus, we apply large matrix operations to compute estimates, standard errors and *p*-values for GWAS with a continuous outcome.

There is a second challenge: reading the data quickly enough from a disk into computer memory. A key issue is to rearrange them in such a way that arbitrary blocks of SNPs (containing all individuals) can be accessed very quickly. We show how to pre-process data for this goal.

The bottom line is that a GWAS for one million SNPs and 10k individuals can be done on an average notebook computer within 15 minutes. This is the time needed for pure computations. Accounting for the time needed to load the data, the whole time of the analysis increases to 25 minutes.

Semi-parallelization of GWAS with a binary outcome is more difficult. Parameters in logistic regression are estimated via maximum likelihood, which unlike the least squares approach is an iterative procedure. However, we were able to find an approximate way to provide odds ratio for the SNP effect using semi-parallel computations.

The paper is written in a tutorial-like manner. We gradually extend the complexity of the problem, showing step by step how to speed up computations using simple tricks in R. Also the goal is not to present a package (there is none) but to introduce a new way of thinking about large-scale GWAS computation and to present and provide code that anyone can easily integrate into existing systems.

## Implementation

### Data, real and simulated

A GWAS is based on very large numbers of SNPs, for many thousands of individuals, leading to very large data files. Observed genotypes generally are coded as the number of reference alleles, 0, 1 or 2. Very efficient storage is possible, using only 2 bits per SNP (per individual). The program PLINK uses this approach to store genotypes in its BED file format. The package SNPstats mimics it for storing SNPs in computer memory. This is quite attractive: 100k SNPs (we use k as shorthand for thousand) for 10k individuals can be stored in a quarter of a Gigabyte.

In large data sets one may expect some values of the traits and/or genotypes to be missing. Typically genotypes with a call rate (percentage of measured genotypes in the sample) below 95% will be removed from the analysis.

The recent GWAS practice is to use genotype imputation. The commonly used MACH[5,6] program does two things: it imputes missing SNPs within genotyped markers and predicts untyped markers. The result of imputation is the expected dose, a non-integer number between 0 and 2. In this case more room is needed to store the values. Actual files with imputation results are then much larger. Those that MACH produces are ASCII-code files, using six positions per number (with three decimals). In principle a more compact representation is possible. There is no need to be precise and by multiplying the dose by 100 we can store an integer between 0 and 200 in one (unsigned) byte. This is four times larger than for raw genotypes.

In our experience, SNP data are stored in such a way that all SNPs for one individual form one record. We call this structure “row per person”. It is useful for random reading of (blocks of) individuals, but selection of certain (blocks of) SNPs is time consuming. Essentially one has to read the complete records for all persons and keep only the required selection of SNPs. This has to be repeated for every block of SNPs one considers.

It is much more attractive to have each record represent one SNP, as measured for all individuals (“row per SNP”). To achieve this, given a “row per person” organization, is an important part of the enterprise. It would not be an issue if all data would fit into fast random-access memory, but this is usually not the case, as we are talking of 10 to 100 Gb.

There is no need for real data when discussing computation times. Instead we simulate genotypes as random numbers from a uniform distribution between 0 and 2. Phenotypes and covariates are simulated as independent variables coming from a standard normal distribution. In our simulations we set the sample size to a typical GWAS scenario, namely 10K individuals. The number of simulated SNPs is 1000, which is determined by the available RAM.

### Semi-parallel computations

In this section we present semi-parallel computation, using the R programming language as the vehicle for implementation (R version 2.15). We report computation speeds, as achieved on a single PC running Windows XP on an Intel E8400 (3.00 GHz) with 3.2 GB of RAM. We report user times provided by the R function proc.time. User time is defined as the CPU time charged for the execution of user instructions of the calling process.

A simple benchmark for comparing to other computers is the time needed for the singular value decomposition (SVD) of a 1000 ×1000 random matrix. For our computer it is 5 seconds in R software.

Our goal is to report computation times in a standardized way, such that they can be easily recalculated for different numbers of individuals and/or SNPs. Computation time for GWAS is linear in sample size and in the number of investigated SNPs. We express speed in “sips” standing for “snp-individual per second”. It is obtained by dividing the product of the numbers of individuals and SNPs by the time needed for a computation. Conversely, if one divides the product of the number of individuals and the number of SNPs by speed, one obtains the number of seconds needed for a job. One should keep in mind that due to the imprecision of proc.time and its variability from run to run, the calculated times/speeds are only approximate. They are provided to assess the order of magnitude of the times gained in computations. Because of the size of the numbers, we will exclusively use Msips, meaning one million sips.

### Regression without additional covariates

Let the (continuous) phenotype be given as a vector *y* of length n and the states of *m* SNPs as the *n*×*m* matrix *S*. A single column of *S* will be denoted as *s*. Unless stated otherwise, we use the same symbols for the R variables. To detect potential genetic effects on *y*, the linear model

(1) y=α+βs+ϵ

is fitted for each SNP and the size of $\hat{\beta }$ is evaluated. Generally the estimated effects are disappointingly low. A culture has grown in which one searches for low (Bonferroni corrected) *p*-values, using large to very large sample sizes. To compute *p*-values we need standard errors, but we will not consider them until the model with covariates has been discussed. A straightforward way to fit the model (1) is to use the function lm repeatedly:

The reported speed is 0.8 Msips, meaning that for this sample size we can test 80 SNPs per second. For 2.5 M SNPs we would need almost 9 hours. In the code above we included the statements used to compute processing times and speed. They will not be shown in the upcoming examples. A faster alternative to lm is lsfit, recording a speed of 5.3 Msips.

For this simple regression problem, we know how to compute the slope explicitly:

(2) $$\hat{\beta }=\frac{\overset{n}{\underset{i=1}{\sum }}(s_{i}-\bar{s})(y_{i}-\bar{y})}{\overset{n}{\underset{i=1}{\sum }}{\left(s_{i}-\bar{s}\right)}^{2}}.$$

This is implemented in the following code, which increases the speed to 26 Msips.

So far, we considered cases where the analysis is implemented in a loop, for one SNP at a time. However, loops are inefficient and it is better to vectorize the computations. That leads us to our first *semi-parallel* algorithm. In the previous code fragment we took each column of the SNP matrix, to center it and compute its inner product with centered *y*, $\tilde{y}$. If we center all columns at once using the function scale we obtain the whole vector $\hat{\beta }$ without using loops. However, when running the code below

we get an unpleasant surprise: the speed drops to 19 Msips. It turns out that scale is a very slow function. We were able to avoid it when we did the calculations ourselves and achieved a speed of 45 Msips.

Centering columns of the SNP matrix is actually not necessary. We can rewrite the numerator of the Equation (2) as

$$\overset{n}{\underset{i}{\sum }}{\tilde{y}}_{i}(s_{i}-\bar{s})=\overset{n}{\underset{i}{\sum }}{\tilde{y}}_{i}s_{i}-\bar{s}\overset{n}{\underset{i}{\sum }}{\tilde{y}}_{i}=\overset{n}{\underset{i}{\sum }}{\tilde{y}}_{i}s_{i}.$$

Similarly, we can show that the denominator of (2) can be rewritten as

$$\overset{n}{\underset{i}{\sum }}{\left(s_{i}-\bar{s}\right)}^{2}=\overset{n}{\underset{i}{\sum }}s_{i}^{2}-n{\left(\bar{s}\right)}^{2}$$

This leads to the following code, running at 90 Msips.

This means that to analyze a GWAS with 2.5 mln of SNPs and 10k individuals around 5 minutes are needed. However, this is for an unrealistic scenario, without covariates. Also, we have not calculated the *p*-values yet. We will now discuss the needed extensions.

### Regression with covariates

To handle covariates in a matrix *X*, we extend the model (1) to

(3) y=βs+Xγ+ϵ,

where it has been assumed that *X* contains a column of ones, to cater for an intercept. A straightforward application of this model uses lm in a loop, as shown below.

**Figure 1:**
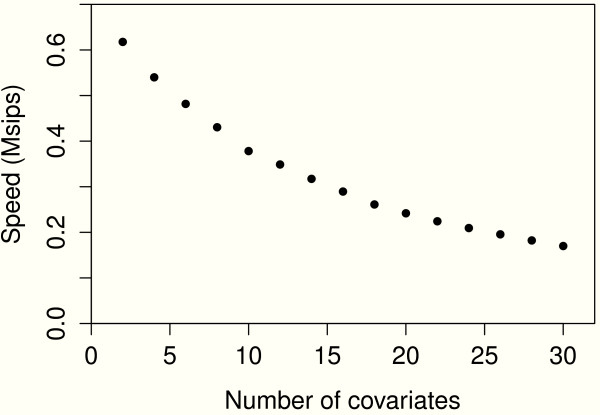
**Speed versus number of covariates.** The plot shows the relationship between the speed of the computations using lm function in R and the number of the covariates in the linear regression model.

Of course the speed will now depend on the number of covariates. This relation is shown in Figure 1. We can also repeatedly apply function lsfit in the following manner.

For 10 covariates, speed is equal to 1.17 Msips. It is again faster than lm, but the whole GWA scan for 2.5 mln SNPs and 10K individuals would still take around 6 hours (18 hours for lm). Shabalin [9] has briefly discussed how to deal with one covariate in an efficient way. The main idea is to orthogonalize the response and the predictor of interest (here the SNP) with respect to that covariate. We derived it for the general case with *k* covariates (Additional file 1: Appendix). The transformed variables are given by the equations:

(4) $$s^{*}=s-X{\left(X^{T}X\right)}^{-1}X^{T}s$$

(5) $$y^{*}=y-X{\left(X^{T}X\right)}^{-1}X^{T}y$$

Assuming that the intercept was included in the matrix of covariates, the model is now simplified to

(6) $$y^{*}=\beta s^{*}+\epsilon$$

It is important to calculate *y*^∗^ and *s*^∗^ efficiently. If we multiply the matrices in order as they appear in (4) and (5), R will encounter memory problems when working with *n*×*n* matrix. A code fragment for well-organized calculations is shown below.

Speeds are 45, 25, 13 Msips for 2, 10 and 30 covariates respectively, about 70 times faster than using lm.

### Standard errors and *p*-values

The variance for the estimated $\hat{\beta }$ in model (6) is given by

(7) $$\hat{\text{var}}(\hat{\beta })={\hat{\sigma }}^{2}{\left(s^{*T}s^{*}\right)}^{-1}.$$

The error variance is estimated by

(8) $${\hat{\sigma }}^{2}=\frac{\text{RSS}}{n-k-2},$$

where the residual sum of squares (RSS) is calculated as

(9) $$\text{RSS}={\left(y^{*}-s^{*}\hat{\beta }\right)}^{T}(y^{*}-s^{*}\hat{\beta })=\overset{n}{\underset{i}{\sum }}y_{i}^{*2}-{\hat{\beta }}^{2}\overset{n}{\underset{i}{\sum }}s_{i}^{*2}$$

Note that in the degrees of freedom we have accounted for the removed covariates, although this usually will be of minor influence. The standard errors of $\hat{\beta }$ and logarithm of the *p*-values can be calculated with the code below.

The calculation of the *p*-values assumes, given the large sample size, that the test statistic has a normal distribution. We used the lower tail of the normal distribution to calculate the *p*-values. It is not advisable to use the textbook definition 2 * (1-pnorm(b / err)), because it suffers from severe rounding errors.

We make a final comparison of speed between lm, lsfit and our fast computations. Standard errors and *p*-values are not included in the lsfit function, but are easily obtained using ls.print procedure. The results, for different numbers of covariates, are provided in the Table 1. We see that the standard function lm is the slowest, but the computational benefits of lsfit decrease for the cases with many covariates. Using semi-parallel computations, we can do a GWAS 61 times faster than with lm for no covariates and 75 times faster for a model with 30 covariates. A GWA scan for 10K individuals, 2.5M SNPs and 10 covariates can be now done within 20 minutes.

**Table 1:** **Speed in Msips for linear model (estimates, standard errors and*p*-values) with*k* covariates for the functions ls, lsfit and semi-parallel (SP)**

**k**	lm	lsfit	**SP**
0	0.70	3.0	43.0
2	0.60	2.4	43.0
10	0.40	1.0	25.0
30	0.16	0.32	12.0

We tested our codes on a another PC with Intel Xeon(R) X5550, 2.67 GHz, 24 GB of RAM and the 64 bit version of R. This machine was around 1.4 times faster than our PC. However, the ratios of the speeds remained similar. Semi-parallel approaches is 60–80 times faster than looping function lm.

### Missing genotypes

The semi-parallel algorithm does not allow missing values. A single NA in either a phenotype vector or a SNP matrix will result in NA in the vector of estimates.

Incomplete phenotypes are easy to handle. We can exclude those individuals from the whole analysis. Missing genotypes are more problematic. In general missing data can be handled using weighted least squares estimation, taking as weights 0 and 1 for missing and available observation. However the weights will vary for different SNPs and semi-parallel approach for the model with covariates cannot be applied anymore.

**Figure 2:**
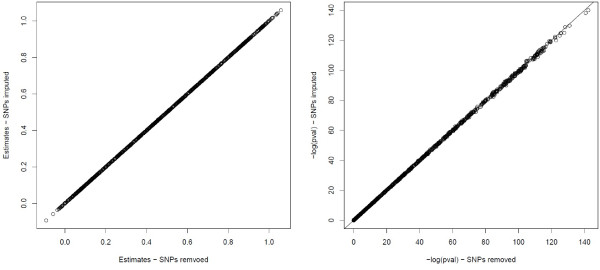
**Imputation of missing SNPs using sample mean.** The plot displays the effect of the imputation of the missing SNPs using sample mean on the estimates and the *p*-values. The call rate is set to 95%.

We propose a very simple solution for the analysis of a GWAS with incomplete SNPs by imputing the missing SNP values with the sample mean of the observed genotypes. Our simulations show that for large sample size (thousands of individuals) and even 5% missing genotypes no substantial precision is lost (Figure 2).

### Logistic regression

When there is an interest in association between a binary outcome and SNPs, logistic regression is needed. The model without additional covariates is given by

(10) $$\log\left(\frac{p}{1-p}\right)=\beta_{0}+\beta_{1}s,$$

with *p* representing probability of “success”.

No closed-form expression exists for the coefficient values. Instead, the (logarithm of the) likelihood function is maximized using iterative procedures like Newton-Raphson or Fisher scoring. The maximization begins with a tentative solution which is iteratively improved until convergence. In R the straightforward way to fit a logistic model is to call a function fitting generalized linear model specifying proper outcome distribution (binomial) and link function (logit). It can be easily done using the code:

The speed is 0.2 Msips which is four times slower than fitting a regression model to a continuous outcome.

A relation exists between maximum likelihood estimation using Fisher scoring and weighted least squares estimation [10]. Maximum likelihood equations for the (*t*+1)-th iteration can be written as

(11) $$(X^{T}W^{\left(t\right)}X)\beta^{\left(t+1\right)}=X^{T}W^{\left(t\right)}z^{\left(t\right)},$$

where *z* is a “working variable” given by

(12) $$z_{i}^{\left(t\right)}=\log\left(\frac{p_{i}^{\left(t\right)}}{1-p_{i}^{\left(t\right)}}\right)+\frac{y_{i}-p_{i}^{\left(t\right)}}{w_{i}^{\left(t\right)}}$$

and where *W*^(*t*)^ is diagonal matrix with elements $p_{i}^{\left(t\right)}(1-p_{i}^{\left(t\right)}).$ Every update of *β* involves solving a weighted least squares problem with updated weight matrix. This process is called iteratively reweighted least squares. The covariance matrix is given by

(13) $$\hat{\text{cov}}({\hat{\beta }}^{\left(t+1\right)})={\left(X^{T}W^{\left(t\right)}X\right)}^{-1}.$$

In case of a model without additional covariates the estimated SNP effect and the standard error are given by

(14) $$\hat{\beta_{1}}=\frac{\underset{i}{\sum }w_{i}(z_{i}-z_{w})(s_{i}-s_{w})}{\underset{i}{\sum }w_{i}{\left(s_{i}-s_{w}\right)}^{2}}$$

(15) $$\hat{\text{var}}(\beta_{1})=\frac{1}{\underset{i}{\sum }w_{i}{\left(s_{i}-s_{w}\right)}^{2}},$$

where *z*_*w*_ and *s*_*w*_ are weighted means defined as $\underset{i}{\sum }w_{i}z_{i}/\underset{i}{\sum }w_{i}$ and $\underset{i}{\sum }w_{i}s_{i}/\underset{i}{\sum }w_{i}$ respectively.

It is not possible to semi-parallelize logistic regression computations to provide an exact solution, because in principle the weights are different for each SNP. However, effects found in GWAS are usually of modest size, with a median odds ratio of 1.33 and only a few odds ratios exceeding 3.00 [11]. This means that probabilities predicted by a model without a SNP will not change much once SNP is included to the model. We can do semi-parallel computations approximately using weights from the model without SNP $\left(\tilde{w}\right)$ as starting values and updating the solution for *β*_1_ by one iteration. Note that in case of no other covariates we have to fit the model with only intercept. The predicted probabilities are the same for every individual and so are the weights. In that special case the weighted mean is equal to the arithmetic mean and (14) reduces to (2). The computations can be easily done in R using the code:

The speed is 55 Msips which is 275 faster than using glm.

**Figure 3:**
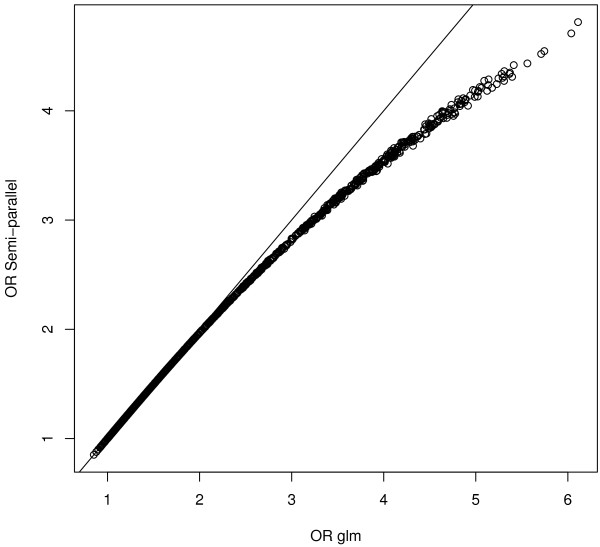
**Logistic OR.** The plot displays odds ratios from the “standard analysis” with glm function (x-axis) versus corresponding odds ratios from semi-parallel approach (y-axis).

**Figure 4:**
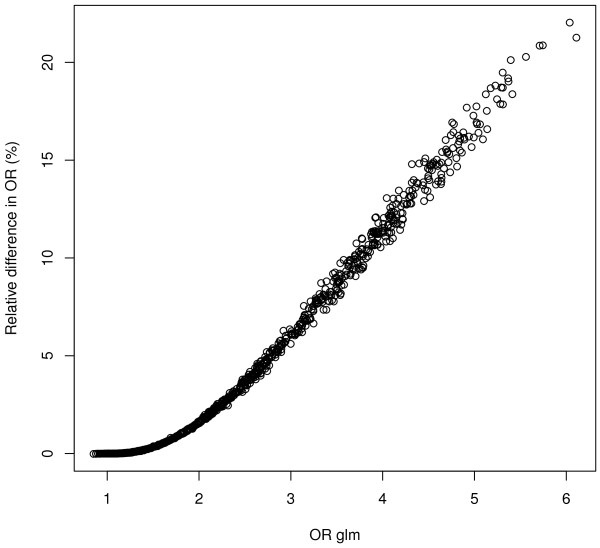
**Logistic Relative differences.** The plot shows the relative difference (in %) in odds ratios between glm and semi parallel approach.

**Figure 5:**
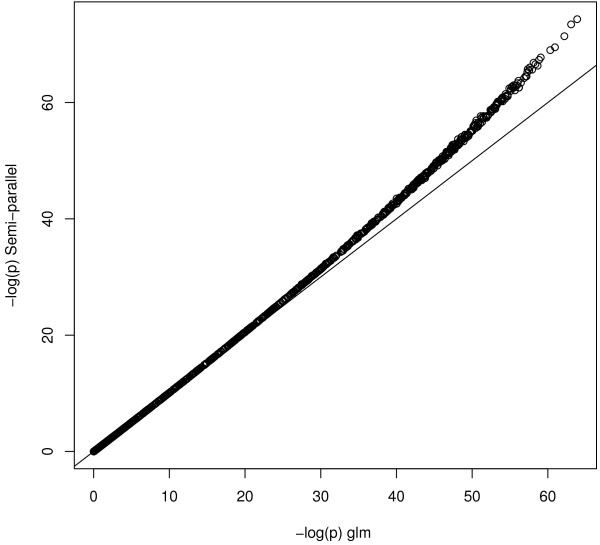
**Logistic P values.** The plot displays the -log(pval) for the SNP effect from the ‘standard analysis’ using glm (x-axis) versus corresponding -log(pval) from semi-parallel approach (y-axis).

Obviously, the quality of approximation of the weights from the model without the SNP depends on the magnitude of *β*_1_. We conducted a small simulation experiment exploring an effect of a true odds ratio on the accuracy of estimation in semi-parallel approach. We simulated 1000 logistic regression models in which true OR was a random number between 1 and 5. We calculated the relative difference of the odds ratios estimated by glm function and semi-parallel approach. The relative difference is increasing monotonically and non-linearly with the correctly estimated OR (**Figure 3 and Figure 4: Logistic OR and Logistic relative differences**). The semi-parallel approach underestimates the OR by 0.1% for OR = 1.33, by 6% for OR = 3 and by 17% for OR = 5. This result was independent from the sample size. Additionally, the *p*-values in semi-parallel approach were slightly too significant, but the difference was observed only for the - log10(*p*) above 25 (for the sample size 2000, **Figure 5: logistic pvalues**). We do not find those observations worrisome in a typical GWAS scenario. However, we leave it up to the user to additionally fit the glm model to a selection of the most promising SNPs.

Dealing with covariates in semi-parallel logistic regression follows the same reasoning as in linear regression, but taking the weight matrix into account. The equations for transformed SNP (*s*^∗^) and *z*^∗^ are

(16) $$s^{*}=s-X{\left(X^{T}\text{WX}\right)}^{-1}X^{T}\text{Ws},$$

(17) $$z^{*}=z-X{\left(X^{T}\text{WX}\right)}^{-1}X^{T}\text{Wz},$$

where again *X* is a matrix of covariates including an intercept. The weight matrix *W* is replaced with $\tilde{W}$ coming from the model without SNP. After the transformation the solution for SNP effect and the standard error are given then by

(18) $$\hat{\beta_{1}}=\frac{\underset{i}{\sum }w_{i}z_{i}^{*}s_{i}^{*}}{\underset{i}{\sum }w_{i}s_{i}^{*2}},$$

and

(19) $$\begin{array}{ll} \hat{\text{var}}(\beta_{1})=\frac{1}{\underset{i}{\sum }w_{i}s_{i}^{*2}}. & \; \end{array}$$

Noting that in this case weights are different for every individual, we can compute the solution by running the following R code:

Comparisons of speeds between semi-parallel approach and glm for different number of covariates are presented in Table 2. The speed gains are between 80 times for the model with 30 covariates and 170 times for the model with 10 covariates, making the efficiency even larger than in linear regression.

**Table 2:** **Speed in Msips for logistic model (estimates, standard errors and*p*-values) with*k* covariates for the functions glm and semi-parallel (SP)**

**k**	**glm**	**SP**
1	0.2	20.0
10	0.1	17.0
30	0.1	8.0

## Organization of the SNP data

Our semi-parallel algorithms substantially reduce computation times. However before we can apply our algorithm we need to load the data into computer memory. Of course this always is an issue, but not really critical when computations are slow.

We assume that we have limited memory available, say 2 to 4 Gb. With 64 bit operating systems, 64 bit R and expensive hardware, it is possible to build a system that can have all data in memory. We do not expect the reader to be that lucky. Instead we assume that we will read in blocks of SNPs of reasonable size.

Data loading entails not only CPU but also I/O times. That is why in this section we only focus on the elapsed time provided by proc.time. This is the clock time measured from the start of the operation until its completion.

We propose different solutions depending on the type of the genotypes we are dealing with (observed or imputed). We show how to efficiently deal with PLINK data formats. For imputed dosage (MACH) files, we discuss what the difficulties are when loading in the structure necessary to apply fast computation algorithm. We describe two R packages: **ff**[12], **ncdf**[13] which we found most useful to tackle this problem.

### Observed genotypes in PLINK format

As an example, we utilized a PLINK BED file that we encountered at our institution. This file stores around 42000 SNPs on chromosome 1 measured for about 6000 persons. Some values were coded as missing. We can easily read a PLINK BED file into R using the function read.plink implemented in the package SNPstats [14].

This will store the genotypes in a SnpMatrix raw format. It is a very efficient storage scheme, using only 2 bits for each element of the SNP matrix. It takes around 10 seconds to load the data. Of course, this can be done only if the matrix fits in memory, but that is no problem here. Another useful feature of the SnpMatrix object is that the indexing operator returns a matrix. Having a SnpMatrix object, we can extract blocks of SNPs to a floating point matrix. The maximum allowed size of the block depends on the available memory and the operating system.

Another problem that we have to deal with are missing genotypes, but in the previous section we proposed a simple strategy to overcome this problem. In the code given below we assume that the missing values are coded as 3 and that the threshold for the call rate is 5%.

Once “bad” SNPs have been removed we can apply a fast computation algorithm. We analyzed a model with correction for 25 covariates. The association scan for our example data file was finished within one minute.

### Imputed genotypes in MACH format

MACH files are larger than PLINK files and may include hundreds of thousands of SNPs written as “row per person” in text files. On a computer without large amount of RAM we will not be able to read into R the whole data file. We have to work with blocks of SNPs. The “row per person” structure is very inefficient if we want to read only a group of SNPs (say 1000) for all individuals. Having a transpose of it, so the “row per SNP” would make it possible for function scan to create a matrix with a block of SNPs for all persons. But even then, reading 1000 SNPs for 10000 individuals takes around 13 seconds. For a genome with 2.5 mln SNPs we would need around 9 hours just to bring the data into R.

There are other, faster ways to deal with large data files in R. One possibility is to work with binary files. Saving and reading binary files is easily done using writeBin and readBin. However, those files work on vectors. This is not an optimal solution for us. Saving all the genotypes for individuals sequentially will not allow us for an easy access to the blocks of SNPs later on.

There are several packages available which deal with array oriented binary files. We will discuss here **ncdf** and **ff** which we found the most useful. The Network Common Data Form (netCDF) are commonly used in meteorology and oceanography. Recently the R package **ncdf** was released to support this data format [13]. First, the MACH data files have to be saved into a **ncdf** object. Our experiments showed that it is most efficient to work with blocks of SNPs and individuals. We will denote bsx and bsy as block size for individuals and SNPs respectively. Number of blocks will be denoted as nbx and nby. We need to define dimensions and variables of the **ncdf** object.

For the specified variables, a netCDF file is created using

If Z is a bsx ×bsy block read by the function scan, the data can be easily stored into **ncdf** file

Saving 100000 SNPs for 6000 persons would take around 45 minutes on our computer. To estimate the complete time, we need to add the time needed for scanning the MACH file (about 15 minutes). To read back the file created above (with the same block sizes) we have to use the following code

Reading goes very fast and is done within 30 seconds.

The package **ff**[12] was created to support memory efficient storage of the large data files. Keeping notation from the **ncdf** example, an **ff** object creation and data storage are done using

After the object is created, the R workspace should be saved. The data saving is faster in **ff** than in **ncdf**. It is linear with the number of individuals’s block for the fixed number of SNPs. We recorded less than a minute necessary to save 100000 SNPs for 6000 persons. To read back the blocks we need to load the saved R workspace. This workspace keeps the pointer to the **ff** file. After that, data reading is very straightforward

The elapsed times for reading are as similar to those of **ncdf**.

## Conclusions

Computations for GWAS were made easy. We have shown that they can be rearranged as large matrix operations performing 60–80 times faster for linear regression and up to 300 times faster for logistic regression. The algorithms can be written in pure R and they do not exceed 20 lines of code.

Fast computations demand fast access to the data and this is actually a harder problem. Not all SNP data fit in memory at the same time. They have to be read in as blocks containing all individuals and selections of SNPs. In practice data are not organized in this way, but as records that contain all SNPs for each individual. We have shown two ways to rearrange data, in a preliminary step, to make fast access possible. Our first solution uses the standardized netCDF file format. It has the advantage that the files can be exchanged easily between computers, operating systems and programming languages. Our second solution uses memory mapped files, as implemented in the package **ff**. It is the fastest solution and it is easy to use, but it is less portable than netCDF.

We believe that we have presented here an attractive solution to computations for relatively large GWAS, on modest hardware, using pure R code. Our algorithms are still “embarrassingly parallel”: it is trivial to divide the task over multiple machines, each working on a different block of SNPs. However, using the package SNOW to exploit multiple processors in one PC, we discovered that it takes so much time to load the data into separate processes that it was not worth the effort.

## Discussion

Using the many processors on modern graphic cards looks like an attractive road to explore. We feel that we are still in a transition phase in which easily accessible libraries for R are not yet available. At the moment of writing this manuscript, most available packages are tied to Nvidia GPUs and needed special installation procedures. We have not yet explored this approach.

A more complicated case of weighting is encountered when one corrects for correlation between individuals. Because the relationship matrix has as many rows and columns as the number of individuals, this poses a real challenge. Several solutions have been proposed, see [15]. More research is needed to determine whether they can be combined with our semi-parallel approach.

GWAS for static phenotypes is only one important issue. Much more challenging are longitudinal data, in which multiple measurements per individual are available. In general the number of measurements varies between persons, as well as the times of observation. One has to use linear mixed models, which entail heavy computation loads. A typical mixed model with 10K observations takes about 1 second, implying a speed of 0.01 Msips, more than 100 times slower than a linear model. The need for fast computations in case of longitudinal data and few approximate procedures have been described in [16]. We are working on algorithms involving large matrix operations for massive fitting of linear mixed models. We have had some successes, but a lot has still to be done. We will report on this subject in due time.

## Availability and requirements

**Project name:** GWASP**Project home page:** https://bitbucket.org/ksikorska/gwasp**Operating systems :** Platform independent**Programming language:** R**Other requirements:** R 2.15 or higher + ncdf and ff packages**Licence:** GPL licence

## Competing interests

The authors declare that they have no competing interests.

## Authors’ contributions

PE designed semi-parallel algorithms. KS designed data reading approaches. PE and KS performed the computations and wrote the manuscript. PG gave useful technical suggestions improving the speed of the algorithms. EL revised the manuscript and gave comments helpful to finalize it. All authors read and approved the final manuscript.

## Supplementary Material

Additional file 1 **Appendix.** The Appendix includes derivation of orthogonal projections used to simplify linear regression model with covariates to simple linear regression. Click here for file
