# Supplementary material for: GWAS on your notebook: fast semi-parallel linear and logistic regression for genome-wide association studies
Source: BMC Bioinformatics. 2013 May 28;14:166. doi: 10.1186/1471-2105-14-166 (PMC3695771; doi:10.1186/1471-2105-14-166)
Supplement: Additional file 1 — Appendix. The Appendix includes derivation of orthogonal projections used to simplify linear regression model with covariates to simple linear regression. [file 1471-2105-14-166-S1.PDF]

APPENDIX

The minimum least squares fit for the model

$$y = \beta s + X\gamma + \epsilon, \tag{1}$$

is obtained by solving the linear system of equations

$$\begin{pmatrix} s's & s'X \\ X's & X'X \end{pmatrix} \begin{pmatrix} \hat{\beta} \\ \hat{\gamma} \end{pmatrix} \begin{pmatrix} s'y \\ X'y \end{pmatrix} \tag{2}$$

for $\hat{\beta}$ and $\hat{\gamma}$. This system is equivalent to the two equations

$$s^T s\hat{\beta} + s^T X\hat{\gamma} = s^T y \tag{3}$$

$$X^T s\hat{\beta} + X^T X\hat{\gamma} = X^T y \tag{4}$$

From equation (3) it follows that

$$\hat{\gamma} = (X^T X)^{-1}(X^T y - X^T s\hat{\beta}). \tag{5}$$

If we insert that into the equation (4) equation we get

$$(s^T s - s^T X(X^T X)^{-1} X^T s)\hat{\beta} = s^T y - s^T X(X^T X)^{-1} X^T y \tag{6}$$

We introduce new SNP variable s^* and new outcome y^* defined as

$$s^* = s - X(X^T X)^{-1} X^T s, \tag{7}$$

$$y^* = y - X(X^T X)^{-1} X^T y. \tag{8}$$

Note that the transformed variables have clear a interpretation. They are part of s and y that is orthogonal to the space spanned by the columns of X, since $X(X^T X)^{-1} X^T s$ and $X(X^T X)^{-1} X^T y$ are the projections of s and y on X.

Now equation (6) has the form

$$s^T s^* \hat{\beta} = s^T y^*. \tag{9}$$

We can easily calculate the following

$$s^{*T} s^* = [s^T - s^T X(X^T X)^{-1} X^T][s - X(X^T X)^{-1} X^T s] = s^T s - s^T X(X^T X)^{-1} X^T s = s^T s^* \tag{10}$$

Similarly we can get that $s^{*T} y^* = s^T y^*$. We can now write the equation (9) as

$$s^{*T} s^* \hat{\beta} = s^{*T} y^*. \tag{11}$$

Therefore, $\hat{\beta}$ is a solution of a new regression model

$$y^* = \beta s^* + \epsilon, \tag{12}$$

with simpler solution (as the new model has no intercept)

$$\hat{\beta} = \frac{\sum_i^N s_i^* y_i^*}{\sum_i^N s_i^{*2}}. \tag{13}$$
